# From Nanoparticle Design to Clinical Translation in Cancer Therapy

**DOI:** 10.3390/ijms27052253

**Published:** 2026-02-27

**Authors:** Jordi Puiggalí

**Affiliations:** Departament de Enginyeria Química, Escola d’Enginyeria de Barcelona Est, Universitat Politècnica de, Catalunya, Av. Eduard Maristany 10-14, 08019 Barcelona, Spain; jordi.puiggali@upc.edu

**Keywords:** nanoparticles, cancer therapy, nanomedicine, drug delivery, targeting strategies, tumor microenvironment, cancer immunotherapy, lipid nanoparticles, polymeric nanoparticles, bioinspired nanomaterials, inorganic nanoparticles, hydroxyapatite, mRNA delivery, translational nanomedicine

## Abstract

Nanoparticle-based strategies have emerged as a versatile and powerful approach for cancer therapy, enabling the integration of material science, molecular biology, and immunology into multifunctional therapeutic platforms. Over the past decade, significant advances in nanoparticle design have expanded their potential beyond passive drug carriers toward systems capable of active targeting, microenvironment-responsive behavior, and immune modulation. This review provides a comprehensive and up-to-date overview of the major nanoparticle platforms developed for cancer treatment, including lipid-based, polymeric, inorganic, and bioinspired nanomaterials, with particular emphasis on their structure–property relationships and biological interactions. We discuss key targeting strategies, spanning passive, active, stimuli-responsive, and cellular or immune-mediated approaches, and analyze how nanoparticles can overcome biological barriers imposed by the tumor microenvironment, such as abnormal vasculature, dense extracellular matrix, hypoxia, and immunosuppression. Special attention is given to nanoparticle-enabled cancer immunotherapy, including vaccine delivery, mRNA–lipid nanoparticle systems, and combination strategies that integrate immunotherapy with conventional treatments. Finally, we critically examine safety, toxicity, and translational challenges that continue to limit the clinical impact of cancer nanomedicine, highlighting the importance of biologically informed design, manufacturing robustness, and regulatory considerations. By synthesizing current advances and identifying emerging trends, this review aims to provide a framework for the rational development of next-generation nanoparticle-based cancer therapies with improved clinical relevance.

## 1. Introduction

Over the past three decades, nanomedicine has been extensively explored as a strategy to improve cancer therapy by enhancing drug delivery, reducing systemic toxicity, and enabling novel therapeutic modalities. Cancer remains one of the leading causes of morbidity and mortality worldwide. According to recent global estimates, more than 19 million new cancer cases and approximately 10 million cancer-related deaths were reported in 2020, with projections indicating a substantial increase over the coming decades due to population aging and demographic growth. Beyond mortality, cancer represents a major contributor to disability-adjusted life years (DALYs), reflecting the combined burden of premature death and years lived with disability. Recent Global Burden of Disease analyses estimate that cancer accounts for hundreds of millions of DALYs globally, underscoring its profound socioeconomic and healthcare impact [1,2]. These figures highlight the urgent need for more effective and safer therapeutic strategies, thereby reinforcing the relevance of advanced nanomedicine approaches in oncology. Early developments were largely inspired by the enhanced permeability and retention (EPR) effect, which suggested that nanoscale carriers could preferentially accumulate in tumors owing to abnormal vasculature and impaired lymphatic drainage. This concept provided a strong initial rationale for nanoparticle-based drug delivery and shaped the design of first-generation anticancer nanomedicines.

Despite this theoretical foundation, the clinical translation of cancer nanomedicine has proven to be considerably more challenging than initially anticipated. Quantitative studies have revealed that only a very small fraction of systemically administered nanoparticles ultimately reaches tumor tissue, underscoring the intrinsic limitations of passive accumulation strategies. In this context, Mitchell et al. [3] proposed a comprehensive framework for engineering precision nanoparticles, emphasizing the critical influence of biological barriers, biodistribution, and rational material design. Similarly, Bhatia and colleagues [4] critically reassessed the field, arguing that many translational shortcomings arise not from the failure of nanotechnology itself but from an oversimplified understanding of tumor biology.

Complementing these perspectives, broader state-of-the-art reviews have mapped the rapidly evolving landscape of cancer nanomedicine. Xu et al. [5] systematically summarized emerging nanotechnological strategies for cancer therapy, spanning diverse material classes and therapeutic modalities, while highlighting the importance of integrating molecular-level understanding with translational considerations. In parallel, Sun et al. [6] emphasized the development of “smart” nanoparticles capable of responding dynamically to tumor-associated stimuli, such as pH gradients, redox conditions, and enzymatic activity, thereby moving beyond static carrier designs.

More recently, increasing attention has been directed toward the intersection of nanotechnology and cancer immunotherapy. Fallatah et al. [7] provided a comprehensive overview of nanoparticle-based approaches to modulate antitumor immune responses, including antigen delivery, immune cell reprogramming, and combination strategies with immune checkpoint inhibitors. In addition, nucleic-acid-based nanomedicines have emerged as a particularly promising therapeutic class. Reviews by Kon et al. [8] and Estapé Senti et al. [9] discussed the potential of mRNA-loaded lipid nanoparticles for cancer therapy and immunotherapy while also addressing key challenges related to delivery efficiency, large-scale manufacturing, and clinical translation.

**Figure 1:**
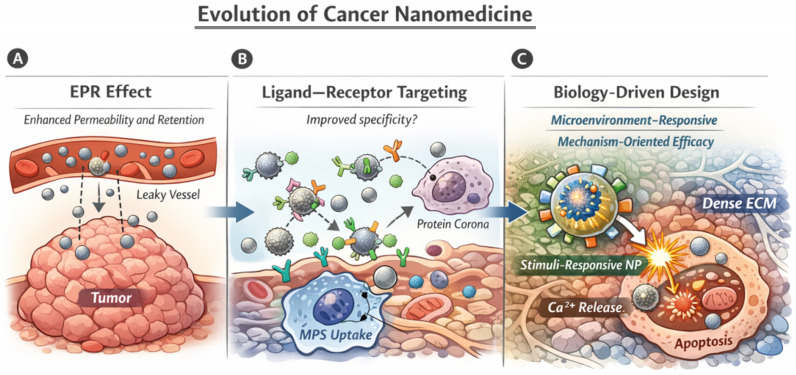
Evolution of conceptual paradigms in cancer nanomedicine: (**A**) Passive accumulation based on the enhanced permeability and retention (EPR) effect. In the classical paradigm, systemically administered nanoparticles (grey spheres) extravasate through abnormally permeable tumor blood vessels (“leaky vessels”) and accumulate passively within the tumor tissue. The tumor is depicted as a relatively homogeneous mass, and nanoparticle transport is mainly driven by passive diffusion. This model assumes that increased vascular permeability and poor lymphatic drainage are sufficient to achieve therapeutic efficacy. (**B**) Active ligand–receptor targeting. In a second-generation approach, nanoparticles are functionalized with targeting ligands (colored surface moieties) designed to bind specific receptors expressed on tumor cells. However, upon contact with biological fluids, nanoparticles rapidly acquire a protein corona, which can mask targeting ligands and alter biological identity. In parallel, a significant fraction of nanoparticles is recognized and cleared by the mononuclear phagocyte system (MPS), represented by macrophage uptake, limiting effective tumor targeting and reducing clinical benefit. (**C**) Biology-driven and microenvironment-responsive nanoparticle design. The current paradigm emphasizes rational nanoparticle design guided by tumor biology and molecular mechanisms. Tumors are depicted as heterogeneous tissues characterized by a dense extracellular matrix (ECM), altered pH, and complex cellular composition. Stimuli-responsive nanoparticles are engineered to interact dynamically with the tumor microenvironment, enabling controlled activation and intracellular effects. As an illustrative example, bioactive nanoparticles capable of releasing calcium ions (Ca^2+^) are shown to induce intracellular stress and apoptosis in cancer cells. This approach prioritizes mechanism-oriented efficacy over passive accumulation, integrating material properties, biological interactions, and therapeutic function.

Collectively, these studies point toward a paradigm shift in cancer nanomedicine, moving from passive, accumulation-driven concepts toward biology-driven and mechanism-oriented nanoparticle design. Rather than focusing solely on tumor accumulation, contemporary strategies increasingly aim to align material properties with tumor heterogeneity, microenvironmental cues, and intracellular transport mechanisms to achieve controlled activation and therapeutic efficacy. This conceptual evolution is illustrated in Figure 1, which summarizes the progression from the classical EPR-based paradigm (Figure 1A), through ligand–receptor targeting strategies constrained by protein corona formation and mononuclear phagocyte system clearance (Figure 1B), to current approaches that explicitly integrate tumor biology, microenvironmental responsiveness, and intracellular mechanisms into nanoparticle engineering (Figure 1C).

Within this emerging framework, bioactive and biodegradable inorganic nanoparticles, such as hydroxyapatite-based nanoplatforms, have gained renewed interest due to their intrinsic biological activity and capacity to modulate intracellular signaling pathways. Their incorporation into the broader landscape of next-generation cancer nanomedicine exemplifies the shift toward materials that do not merely act as passive carriers but actively participate in therapeutic mechanisms, as conceptually depicted in Figure 1C.

**Figure 2:**
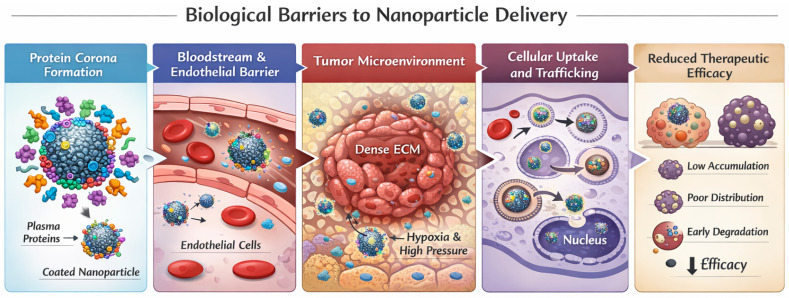
Sequential biological barriers limiting effective nanoparticle delivery to solid tumors. This schematic illustrates the major biological barriers that nanoparticles encounter following systemic administration, which collectively restrict efficient tumor accumulation and therapeutic efficacy. Protein corona formation: Immediately upon exposure to biological fluids, nanoparticles adsorb plasma proteins, forming a dynamic protein corona that defines their biological identity. This process alters nanoparticle surface properties, masks intended functionalization or targeting ligands, and critically influences circulation time, biodistribution, and cellular interactions. Bloodstream and endothelial barrier: Nanoparticles circulating in the bloodstream face heterogeneous blood flow and interactions with endothelial cells. Transport across the tumor vasculature is tightly regulated and often occurs through active transcytotic mechanisms rather than passive leakage, limiting extravasation into tumor tissue. A substantial fraction of nanoparticles is simultaneously cleared from circulation or diverted away from the tumor. Tumor microenvironment barrier: Once extravasated, nanoparticles must navigate the complex and heterogeneous tumor microenvironment. Dense extracellular matrix (ECM), elevated interstitial fluid pressure, hypoxia, and abnormal tissue architecture severely restrict nanoparticle diffusion and penetration, resulting in non-uniform intratumoral distribution. Cellular uptake and intracellular trafficking: Following cellular internalization, nanoparticles are commonly sequestered within endosomal and lysosomal compartments. Inefficient endosomal escape, suboptimal intracellular trafficking, or premature degradation can prevent therapeutic payloads from reaching their intended intracellular targets, such as the cytosol or nucleus. Reduced therapeutic efficacy: The cumulative effect of these sequential barriers leads to low tumor accumulation, poor spatial distribution within tumor tissue, and limited intracellular bioavailability, ultimately resulting in reduced therapeutic efficacy despite optimized nanoparticle physicochemical properties. Together, these interconnected barriers emphasize that successful cancer nanomedicine requires rational design strategies that explicitly account for biological complexity across multiple scales, rather than relying solely on passive accumulation or surface-level targeting approaches.

Despite the conceptual advances outlined above, the efficient delivery of nanoparticles to tumors remains severely constrained by a series of biological barriers operating across multiple spatial and temporal scales. Immediately after systemic administration, nanoparticles interact with biological fluids and rapidly acquire a protein corona, which defines their biological identity and critically influences biodistribution and cellular interactions [10]. Subsequent transport across the vascular endothelium is further limited by heterogeneous blood flow and tightly regulated transcytotic mechanisms, rather than simple passive leakage, as demonstrated by intravital imaging studies of nanoparticle entry into solid tumors [11]. In addition, the dense and highly heterogeneous extracellular matrix represents a major obstacle to deep tumor penetration, contributing to the extremely low overall fraction of nanoparticles that ultimately reach tumor cells [12]. Even following successful cellular uptake, nanoparticles frequently encounter inefficient intracellular trafficking, endosomal sequestration, or premature degradation, ultimately reducing therapeutic efficacy. These sequential and interconnected barriers, which collectively determine the in vivo fate of nanomedicines, are schematically summarized in Figure 2, underscoring why rational nanoparticle design must explicitly account for biological complexity across multiple scales rather than relying solely on passive accumulation or ligand-based targeting strategies.

While previous reviews have provided comprehensive overviews of specific nanoparticle platforms, cancer immunotherapy applications, or translational challenges in isolation, the present work adopts a distinct integrative perspective. Rather than cataloguing nanomaterial systems, this review emphasizes the mechanistic interplay between nanoparticle material design, biological barriers, and tumor microenvironment modulation, linking molecular-level architecture to clinically relevant performance constraints. Particular attention is given to responsive and bioactive inorganic platforms, including hydroxyapatite-based systems, as illustrative examples of materials that actively participate in therapeutic mechanisms beyond passive drug delivery. By structurally connecting biological complexity, materials engineering, and translational feasibility within a single framework, this review aims to provide a cohesive design-oriented roadmap for next-generation cancer nanomedicine.

The objective of this review is to provide a comprehensive and mechanistic overview of recent advances in nanoparticle-based strategies for cancer therapy, with a particular emphasis on how rational material design can address the biological barriers that limit in vivo efficacy. Rather than offering an exhaustive catalog of nanomaterials, this article focuses on emerging design principles, molecular mechanisms, and bio–nano interactions that define next-generation cancer nanomedicines. We first discuss the major biological and cellular barriers that govern nanoparticle fate following systemic administration. We then examine recent developments in nanoparticle materials and architectures, including lipid-based, polymeric, inorganic, and bioinspired platforms, highlighting how their physicochemical and biological properties influence therapeutic performance. Particular attention is given to bioactive inorganic nanoparticles, such as hydroxyapatite-based systems, as illustrative examples of materials that actively participate in therapeutic mechanisms. Subsequent sections address advanced targeting strategies, nucleic-acid-based nanotherapies, interactions with the tumor microenvironment, and combination treatment approaches. Finally, we discuss translational challenges, safety considerations, and future perspectives, including the potential role of advanced preclinical models and data-driven design, to outline realistic pathways toward clinically effective cancer nanomedicines. Rather than cataloguing nanoparticle formulations, this review focuses on mechanistic design principles that align material properties with biological constraints and therapeutic objectives.

## 2. Biological Barriers to Nanoparticle Delivery

The limited clinical success of many nanomedicine-based cancer therapies is largely determined by a complex cascade of biological barriers that govern nanoparticle fate following systemic administration. These barriers operate sequentially and across multiple biological compartments, and their combined effects largely explain the discrepancy between promising preclinical outcomes and modest clinical efficacy [4,12]. A mechanistic understanding of these processes is therefore essential for the rational design of next-generation nanotherapeutics.

### 2.1. Protein Corona Formation and Biological Identity

Upon contact with blood and interstitial fluids, nanoparticles rapidly adsorb biomolecules—predominantly proteins—forming a dynamic protein corona that defines their effective biological identity [10]. The composition and evolution of the protein corona depend on nanoparticle size, surface chemistry, charge, and shape, as well as on the local biological environment [13,14]. Importantly, this acquired biological identity often overrides the intended synthetic surface design, influencing cellular recognition, immune activation, and clearance pathways. Several studies have demonstrated that specific corona profiles can promote opsonization and uptake by phagocytic cells, whereas others may confer partial stealth properties, underscoring the central role of corona formation in determining nanoparticle biodistribution and tumor accessibility [15,16].

### 2.2. Vascular Transport and Endothelial Translocation

Transport across the tumor vasculature represents a critical bottleneck for nanoparticle delivery and is increasingly recognized as a highly regulated and dynamic process. Rather than occurring uniformly through passive leakage, nanoparticle extravasation is governed by spatially and temporally variable endothelial transport mechanisms, including active transcytosis pathways that differ across tumor regions and stages of progression [11]. As a result, vascular transport efficiency is strongly influenced by local hemodynamics, endothelial phenotype, and vessel functionality [17,18], leading to pronounced variability in nanoparticle access to tumor tissue even within the same lesion. This functional heterogeneity of the tumor vasculature fundamentally limits the predictability of nanoparticle delivery and challenges design strategies that rely on uniform transport assumptions [19].

### 2.3. Tumor Microenvironment and Extracellular Matrix Constraints

Following extravasation, nanoparticles encounter the tumor microenvironment, where their transport and distribution are further constrained by the complex and spatially heterogeneous organization of the extracellular matrix (ECM). Dense collagen networks, elevated interstitial fluid pressure, and irregular tissue architecture impose physical barriers that severely restrict nanoparticle diffusion and penetration into tumor parenchyma [18,20]. Unlike vascular transport, which is dominated by dynamic endothelial processes, these structural constraints vary across tumor regions and generate steep intratumoral gradients in nanoparticle concentration. Consequently, even nanoparticles that successfully exit the bloodstream often exhibit limited and non-uniform penetration, resulting in heterogeneous therapeutic exposure at the cellular level [21,22].

In addition to structural and mechanical constraints, the tumor microenvironment profoundly influences angiogenesis. Tumor-associated hypoxia and inflammatory signaling promote the overexpression of pro-angiogenic factors such as vascular endothelial growth factor (VEGF), leading to the formation of structurally abnormal and highly permeable blood vessels. However, this neovasculature is typically disorganized, tortuous, and functionally inefficient, resulting in heterogeneous perfusion and regions of hypoxia. Rather than uniformly enhancing nanoparticle extravasation, such abnormal angiogenesis often exacerbates transport limitations by creating spatial variability in vascular permeability and interstitial pressure. Consequently, strategies aimed at vascular normalization or angiogenic modulation have emerged as complementary approaches to improve nanoparticle delivery and intratumoral distribution [23].

### 2.4. Cellular Uptake and Intracellular Trafficking

Even when nanoparticles reach tumor cells, intracellular barriers can severely limit therapeutic efficacy. Cellular internalization generally occurs through endocytic pathways, leading to sequestration in early and late endosomes and, ultimately, lysosomes [24]. Inefficient endosomal escape remains one of the most critical bottlenecks for nanoparticle-mediated delivery, particularly for nucleic-acid-based therapeutics that require cytosolic or nuclear access [25,26]. Furthermore, intracellular degradation, recycling, or exocytosis can further reduce the effective bioavailability of the therapeutic payload, emphasizing the need to consider subcellular trafficking pathways during nanoparticle design.

### 2.5. Implications for Nanoparticle Design

Together, these vascular, microenvironmental, and intracellular barriers highlight that nanoparticle delivery is governed by a cascade of dynamic and context-dependent processes rather than a single limiting step. Importantly, the spatial and temporal variability of these barriers suggests that static nanoparticle designs are inherently insufficient to achieve consistent therapeutic performance in vivo. Instead, these challenges motivate the development of responsive and adaptive nanoparticle systems capable of sensing, responding to, or exploiting biological cues to improve transport, activation, and efficacy, a concept that underpins the next generation of nanoparticle platforms discussed in the following section.

The interconnected nature of these biological barriers, spanning systemic circulation, tumor extravasation, stromal penetration, and intracellular trafficking, is schematically summarized in Figure 2. This multiscale perspective underscores why successful nanomedicine design must address sequential constraints rather than isolated biological events.

## 3. New Generations of Nanoparticles: Materials and Molecular Design

Recent advances in cancer nanomedicine have demonstrated that nanoparticle performance is not solely determined by cargo loading or targeting ligands but is fundamentally governed by material composition and molecular design. Accordingly, nanoparticle platforms are increasingly classified not only by their constituent materials—such as lipids, polymers, inorganic compounds, or bioinspired components—but also by how their physicochemical properties dictate biological interactions, intracellular trafficking, and therapeutic mechanisms of action [5,6]. While lipid-based and polymeric nanoparticles have achieved the highest level of clinical translation to date, particularly for nucleic-acid-based therapeutics, inorganic and bioinspired nanomaterials have gained renewed attention as multifunctional and biologically active systems capable of integrating therapy, imaging, and microenvironmental modulation [12,18].

**Figure 3:**
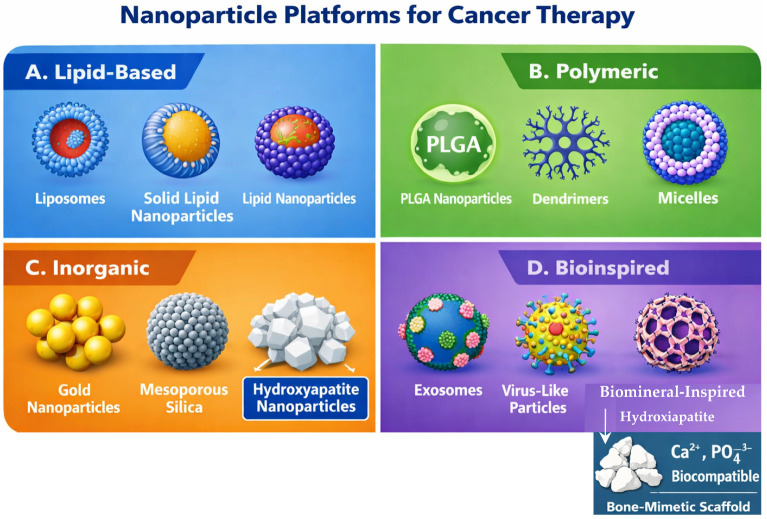
Classification of major nanoparticle platforms for cancer therapy. This figure summarizes the principal classes of nanoparticle-based platforms currently explored for cancer therapy, categorized according to their material composition and biological functionality: (**A**) Lipid-based nanoparticles include liposomes, solid lipid nanoparticles, and lipid nanoparticles, which are widely used for the delivery of small-molecule drugs and nucleic acids due to their biocompatibility, structural versatility, and clinical maturity. (**B**) Polymeric nanoparticles, such as poly(lactic-co-glycolic acid) (PLGA) nanoparticles, dendrimers, and polymeric micelles, offer tunable physicochemical properties, controlled drug release profiles, and flexible surface functionalization. (**C**) Inorganic nanoparticles comprise gold nanoparticles, mesoporous silica nanoparticles, and hydroxyapatite-based nanoparticles. These systems are characterized by high structural stability and multifunctionality, enabling applications in drug delivery, imaging, and combined therapeutic modalities. Hydroxyapatite nanoparticles are highlighted as bioactive and biodegradable inorganic platforms with intrinsic biological interactions mediated by calcium and phosphate ions. (**D**) Bioinspired nanoparticles, including exosomes and virus-like particles, mimic natural biological structures and processes, providing enhanced cellular communication, immune interaction, and targeting potential. Additionally, bone-mimetic hydroxyapatite-based systems are also represented as biomineral-inspired platforms, bridging bioinspired design with inorganic functionality. Together, this classification provides a conceptual framework for understanding how different nanoparticle platforms address distinct therapeutic requirements and biological barriers while illustrating the diversity of material strategies underpinning modern cancer nanomedicine.

To address the biological barriers governing nanoparticle fate in vivo, extensive efforts have been devoted to the development of diverse nanoparticle platforms with tailored physicochemical and biological properties. Rather than relying on a single optimal design, contemporary cancer nanomedicine encompasses a broad spectrum of materials engineered to interact with biological systems in distinct ways. These platforms can be broadly classified according to their material composition and biological functionality, including lipid-based, polymeric, inorganic, and bioinspired nanoparticles (Figure 3). Each class exhibits unique advantages and limitations with respect to drug loading capacity, stability, biodegradability, immune interaction, and translational feasibility [3,4].

Beyond broad material categories, the evolution of cancer nanomedicine is increasingly driven by molecular-level design strategies that enable nanoparticles to respond dynamically to biological cues. Rather than functioning as static delivery vehicles, next-generation nanoparticles are conceived as adaptive systems whose structural integrity, surface properties, or biological activity can change in response to endogenous or exogenous stimuli. This paradigm shift reflects growing awareness that successful delivery and therapeutic efficacy depend on context-dependent interactions with heterogeneous and evolving biological environments.

At the molecular scale, nanoparticle behavior arises from the interplay between intrinsic material parameters—including size, shape, surface chemistry, mechanical stiffness, and degradation kinetics—and extrinsic biological factors such as protein corona formation, cellular uptake pathways, and microenvironmental conditions. These parameters collectively govern circulation lifetime, biodistribution, cellular internalization, intracellular trafficking, and, ultimately, therapeutic outcome. Importantly, many of these features are interdependent, such that optimization of a single property often introduces trade-offs elsewhere, underscoring the need for integrative and mechanism-driven design strategies rather than isolated formulation tuning.

Responsiveness to tumor-associated stimuli represents one of the most actively explored approaches to enhance therapeutic specificity. Solid tumors exhibit a range of physicochemical abnormalities, including acidic extracellular pH, elevated enzymatic activity, hypoxia, redox gradients, and altered ion concentrations. Nanoparticles engineered to sense and respond to these cues can undergo controlled structural rearrangement, surface charge conversion, or cargo release preferentially within the tumor microenvironment or intracellular compartments. Such behavior enables spatial and temporal control over therapeutic action while minimizing off-target effects, addressing key limitations of conventional delivery systems.

Surface chemistry plays a particularly critical role in this context, as it defines the nano–bio interface and directly regulates protein adsorption, immune recognition, and cellular interactions. Among surface-engineering strategies, PEGylation has been widely adopted to enhance colloidal stability and prolong systemic circulation by introducing a hydrated steric barrier that reduces nonspecific interactions. However, PEGylation also exemplifies the inherent “stealth versus interaction” trade-off in nanoparticle design: while effective at reducing opsonization and clearance, excessive surface shielding can impair cellular uptake and intracellular trafficking. Consequently, PEGylation is increasingly regarded not as a fixed formulation element but as a tunable and dynamic design parameter, requiring careful optimization of chain length, grafting density, architecture, and stability under physiological conditions [27].

In parallel, the limitations associated with static surface shielding have motivated the development of adaptive and responsive surface designs. These include cleavable or shedable surface layers that detach in response to tumor-associated stimuli, enabling nanoparticles to transition from a stealth state during circulation to an interaction-competent state at the tumor site. Such strategies exemplify the broader shift toward responsive nanomedicine, in which material design is explicitly aligned with biological context and therapeutic objectives rather than relying on universal solutions [18,27].

Material choice fundamentally influences how responsiveness is implemented. Lipid-based systems can exploit pH-dependent ionization, membrane fusion, or lipid phase transitions; polymeric nanoparticles allow incorporation of cleavable linkers and degradable backbones; inorganic materials often rely on dissolution, redox activity, or ion release; and bioinspired platforms leverage endogenous biological pathways for activation and transport. These diverse mechanisms illustrate that responsiveness is not confined to a single material class but instead emerges from the interplay between chemistry, structure, and biological environment.

An additional challenge in nanoparticle design lies in balancing multifunctionality with translational robustness. While multifunctional nanoparticles capable of simultaneous targeting, imaging, and therapy are conceptually appealing, increasing structural complexity often compromises reproducibility, scalability, and regulatory acceptance. Accumulating evidence suggests that rational simplification and modular design—favoring biodegradable materials and clinically validated components—may offer a more effective path toward translation than maximal functional integration.

Within this framework, hybrid and modular nanoparticle architectures have gained prominence. By combining complementary material classes—such as lipid–polymer hybrids, inorganic–organic composites, or membrane-coated synthetic cores—these systems aim to integrate distinct advantages while maintaining manufacturability and control. For example, lipid–PLGA hybrid nanoparticles developed by Zhao et al. have demonstrated improved mRNA delivery by combining the biocompatibility of lipids with the controlled stabilizing contribution of PLGA, achieving enhanced gene expression in multiple cell models [28]. Importantly, such architectures facilitate systematic investigation of structure–property–function relationships, enabling optimization based on biological performance rather than physicochemical metrics alone.

Taken together, these developments highlight a conceptual transition in cancer nanomedicine from carrier-centric optimization toward biology-guided and mechanism-oriented design. Nanoparticle platforms should therefore be viewed not as universal solutions, but as adaptable tools whose effectiveness depends on alignment with specific therapeutic goals and biological constraints. In the following sections, we examine how lipid-based, polymeric, inorganic, and bioinspired nanoparticles embody distinct design philosophies and address complementary challenges in cancer therapy.

From a translational and manufacturing perspective, nanoparticle design must also account for synthesis yield, batch-to-batch reproducibility, and colloidal stability. Many systems that perform well at laboratory scale face reduced production yields or variability upon scale-up due to incomplete encapsulation, changes in mixing kinetics, solvent exchange, or purification losses. In parallel, aggregation and agglomeration can arise during synthesis, concentration, storage, or after freeze–thaw/lyophilization steps, altering the effective hydrodynamic size distribution, surface charge, and, ultimately, biodistribution and safety. These issues are particularly relevant for high-surface-energy inorganic nanomaterials and soft-matter formulations that rely on kinetically trapped assemblies. Accordingly, robust nanomedicine development increasingly integrates critical quality attributes (e.g., size/polydispersity, zeta potential, encapsulation efficiency, residual solvents) with process controls (e.g., controlled mixing, microfluidic or continuous manufacturing approaches) to ensure scalable production while minimizing aggregation-related performance losses [29,30,31].

### 3.1. Lipid-Based Nanoparticles

Lipid-based nanoparticles represent the most clinically advanced class of nanocarriers for cancer therapy and have played a pivotal role in establishing the translational viability of nanomedicine. This category encompasses conventional liposomes, solid lipid nanoparticles, nanostructured lipid carriers, and lipid nanoparticles (LNPs), which differ in internal organization, lipid composition, and cargo compatibility. Their success is largely attributed to the intrinsic biocompatibility of lipid materials, structural similarity to biological membranes, and extensive prior use in pharmaceutical formulations [32,33].

Early liposomal formulations demonstrated that encapsulation of cytotoxic agents could significantly alter pharmacokinetics and reduce systemic toxicity, as exemplified by doxorubicin-loaded liposomes. However, these first-generation systems relied predominantly on passive tumor accumulation and exhibited limited control over intracellular drug release. Subsequent advances in lipid chemistry and formulation engineering enabled the development of stimuli-responsive and actively targeted liposomes, incorporating pH-sensitive lipids, fusogenic components, or surface-bound ligands to enhance intracellular delivery and therapeutic efficacy [34,35].

PEGylation is particularly central to lipid-based nanoparticles, as PEG–lipids are widely employed to stabilize particles during formulation, prevent aggregation, and modulate pharmacokinetics by controlling protein corona formation and immune recognition. At the same time, lipid nanocarriers clearly illustrate the so-called “PEG dilemma”: PEG layers that improve circulation time and reduce nonspecific interactions can simultaneously decrease productive cell binding and limit intracellular delivery. This trade-off shifts the dominant barrier from systemic exposure to cellular uptake and endosomal trafficking, highlighting PEGylation as both an enabling and limiting factor in lipid-based nanomedicine [27].

These limitations have stimulated extensive efforts toward rational PEG–lipid design, including optimization of PEG chain length, lipid anchor chemistry, and PEG desorption kinetics, as well as the development of responsive PEGylation strategies. In such systems, PEG can be selectively shed or reorganized upon exposure to tumor-associated cues, enabling a transition from stealth behavior in circulation to enhanced cellular interaction at the tumor site. From this perspective, PEGylation functions as a dynamic regulator of nanoparticle–cell interactions rather than a fixed surface modification [27].

A concrete experimental illustration of how PEGylation can be structurally engineered is provided by PEGylated micro- and nanoparticles based on biodegradable poly(ester amides), in which core–shell architectures were characterized using synchrotron radiation-based FTIR microspectroscopy combined with electron microscopy. This approach offers mechanistic insight into the spatial organization of PEG-rich shells and biodegradable cores, supporting rational tuning of degradation behavior, interfacial properties, and biological performance. Such structurally resolved design strategies are highly relevant for lipid-based and hybrid nanocarriers intended for responsive and translational cancer therapy applications [36].

In recent years, lipid nanoparticles have emerged as the dominant platform for nucleic-acid-based cancer therapeutics. LNPs are typically composed of ionizable lipids, phospholipids, cholesterol, and PEG-lipids, forming dynamic assemblies that protect nucleic acids during circulation and facilitate endosomal escape following cellular uptake. Mechanistic studies have shown that ionizable lipids play a critical role in mediating endosomal membrane destabilization through pH-dependent charge transitions, thereby enabling cytosolic delivery of mRNA or siRNA [37,38]. These properties have positioned LNPs as leading candidates for cancer vaccines, gene silencing, and immunomodulatory applications.

Despite their clinical maturity, lipid-based nanoparticles continue to face challenges related to immune recognition and organ-selective biodistribution. In particular, PEGylation has been associated with reduced cellular uptake and, in some cases, anti-PEG immune responses upon repeated administration. In addition, LNP biodistribution remains strongly biased toward the liver due to a polycoprotein-mediated uptake, limiting efficient targeting of extrahepatic tumors [39,40]. These issues have driven the exploration of alternative lipid chemistries, biodegradable ionizable lipids, and ligand-independent targeting strategies to modulate immune interactions and organ selectivity [41,42].

From a translational perspective, lipid-based nanoparticles benefit from well-established large-scale manufacturing processes, including microfluidic mixing and robust quality control, which are critical for clinical and regulatory approval. Nevertheless, growing evidence suggests that further improvements in therapeutic efficacy will require closer integration of lipid nanoparticle design with tumor-specific biology, rather than continued optimization of formulation parameters alone [43,44]. As such, lipid-based systems serve both as a benchmark for clinical feasibility and as a reference point against which emerging nanoparticle platforms can be critically evaluated.

Squalene-based nanoparticles constitute a complementary lipid-derived platform in which therapeutic molecules are covalently conjugated to squalene, allowing spontaneous self-assembly into nanoassemblies with high drug loading efficiency and favorable pharmacokinetic profiles. This strategy has generated extensive preclinical evidence supporting improved anticancer efficacy and reduced systemic toxicity across multiple tumor models [45,46].

### 3.2. Polymeric Nanoparticles

Polymeric nanoparticles constitute a highly versatile class of nanocarriers for cancer therapy, offering extensive control over composition, architecture, and functionalization. Unlike lipid-based systems, polymeric nanoparticles can be engineered with precise molecular weights, defined block structures, and tailored degradation kinetics, enabling fine-tuning of drug loading, release profiles, and biological interactions. Commonly used polymers include poly(lactic-co-glycolic acid) (PLGA), poly(ε-caprolactone) (PCL), poly(ethylene glycol) (PEG)-based copolymers, and a wide range of synthetic and semi-synthetic materials designed for biomedical applications [47,48].

One of the main advantages of polymeric nanoparticles lies in their structural diversity. These systems can be formulated as solid nanoparticles, polymeric micelles, nanogels, dendrimers, or polymersomes, each providing distinct capabilities for encapsulating hydrophobic drugs, proteins, or nucleic acids. In particular, polymeric micelles formed from amphiphilic block copolymers have been extensively investigated for solubilizing poorly water-soluble anticancer drugs and improving their pharmacokinetic profiles [49,50]. Typical systems are based on diblock or triblock copolymers composed of a hydrophilic segment, most commonly poly(ethylene glycol) (PEG), and a hydrophobic or stimuli-responsive block that drives self-assembly into core–shell nanostructures in aqueous environments.

Representative examples include PEG-b-poly(histidine), PEG-b-poly(β-amino ester), and PEG-b-poly(caprolactone)-based copolymers, which form stable micelles at physiological pH but undergo structural rearrangement or disassembly under mildly acidic conditions. Such pH sensitivity is particularly relevant in the context of cancer therapy, as the extracellular tumor microenvironment and intracellular endo/lysosomal compartments exhibit lower pH values compared to healthy tissues. Protonation of ionizable groups within the hydrophobic block can destabilize the micellar core, triggering drug release selectively at the tumor site or following cellular internalization [51,52,53].

Beyond simple drug solubilization, pH-responsive polymeric micelles provide a biologically informed mechanism for controlled intracellular delivery. Micelle destabilization in acidic endosomes can facilitate endosomal escape and enhance cytosolic drug availability, thereby increasing therapeutic efficacy while minimizing systemic toxicity. In this regard, block copolymer design at the molecular level—including block length, pKa of ionizable moieties, and micelle stability—plays a decisive role in dictating biological performance [49,54].

Collectively, these studies illustrate how polymeric micelles can be engineered not only as passive carriers but as dynamic nanostructures that respond to pathological cues and actively participate in the intracellular delivery process, reinforcing their relevance within the broader framework of responsive nanoparticle design. Similarly, dendrimers offer highly branched, monodisperse architectures with multiple functional groups that can be exploited for multivalent drug conjugation and targeting strategies.

Polymeric nanoparticles have also enabled the development of stimuli-responsive delivery systems that exploit tumor-associated cues such as acidic pH, redox gradients, enzymatic activity, or reactive oxygen species. By incorporating cleavable linkers or environmentally sensitive polymer segments, these nanoparticles can undergo controlled disassembly or drug release specifically within the tumor microenvironment or intracellular compartments [55,56]. Such responsiveness provides an additional layer of control beyond passive delivery, potentially enhancing therapeutic selectivity and reducing off-target toxicity.

Despite these advantages, polymeric nanoparticles face several challenges that have limited their clinical translation. Complex synthesis routes, batch-to-batch variability, and difficulties in large-scale manufacturing can hinder reproducibility and regulatory approval. Moreover, polymer degradation products and surface chemistry can influence immune recognition and biodistribution in unpredictable ways, necessitating careful evaluation of long-term safety and clearance [12,57]. In the context of nucleic acid delivery, polymeric vectors often exhibit lower transfection efficiency and higher cytotoxicity compared with lipid-based counterparts, particularly due to insufficient endosomal escape or polymer-associated toxicity [58,59].

From a design perspective, recent efforts have focused on simplifying polymer architectures, integrating biodegradable and bioresorbable components, and combining polymeric systems with other material classes to create hybrid nanoparticles. These strategies aim to balance the molecular precision and multifunctionality of polymeric carriers with the translational robustness required for clinical application [3,5]. As such, polymeric nanoparticles continue to serve as an important platform for exploring advanced delivery concepts, while also highlighting the trade-offs between design complexity and clinical feasibility in cancer nanomedicine.

### 3.3. Inorganic Nanoparticles

Inorganic nanoparticles represent a broad and multifunctional class of nanomaterials in cancer nanomedicine, characterized by their well-defined structures, physicochemical robustness, and unique optical, magnetic, or bioactive properties. Representative platforms include gold nanoparticles, iron oxide nanoparticles, mesoporous silica nanoparticles, calcium-based materials, and metal–organic frameworks (MOFs). Unlike lipid-based or polymeric systems, inorganic nanoparticles often contribute actively to therapeutic mechanisms, enabling combinations of drug delivery, imaging, photothermal or magnetic therapies, and modulation of the tumor microenvironment [60,61].

Despite extensive preclinical exploration, the clinical translation of many inorganic nanoparticle platforms has been limited by concerns related to long-term persistence, insufficient biodegradability, and incomplete clearance from biological tissues [12]. These limitations have prompted increasing interest in bioactive and biodegradable inorganic materials whose degradation products are physiologically tolerated and whose interactions with cells are intrinsically biologically relevant.

Among inorganic nanomaterials, metal oxide nanoparticles with magnetic properties have attracted sustained interest for cancer diagnosis and therapy. Iron oxide nanoparticles, particularly magnetite (Fe_3_O_4_) and maghemite (γ-Fe_2_O_3_), are the most extensively studied magnetic platforms due to their superparamagnetic behavior, chemical stability, and relative biocompatibility. These nanoparticles have been widely explored as contrast agents for magnetic resonance imaging, as well as for magnetically guided drug delivery and magnetic hyperthermia-based cancer therapy [60]. Their ability to generate localized heat under alternating magnetic fields enables minimally invasive tumor ablation while sparing surrounding healthy tissues [62,63]. The magnetic performance of these nanoparticles is strongly dependent on their size, morphology, and crystalline structure, which directly influence coercivity, magnetic saturation (Ms), and heating efficiency under alternating magnetic fields. Superparamagnetic iron oxide nanoparticles (SPIONs) below a critical size typically exhibit negligible coercivity and remanence, minimizing aggregation after field removal and improving biocompatibility. In contrast, larger or morphologically anisotropic particles can display enhanced magnetic anisotropy, leading to increased specific absorption rates (SARs) during hyperthermia treatments. Recent studies have demonstrated that tailoring nanoparticle morphology—such as cubic, rod-shaped, or nanoflower architectures—enables modulation of magnetic domain structure and anisotropy, thereby optimizing therapeutic heating efficiency and imaging contrast [64,65,66]. These findings underscore that magnetic nanoparticle design must integrate structural control at the nanoscale with magnetic physics considerations to achieve predictable therapeutic outcomes. Beyond thermal effects, magnetic nanoparticles can be functionalized with polymers, targeting ligands, or therapeutic cargos, enabling multifunctional theranostic systems that integrate imaging, targeting, and therapy within a single inorganic platform. However, challenges related to aggregation, long-term accumulation, and clearance remain, underscoring the importance of surface engineering and biodegradable design strategies [67,68].

Gold nanoparticles represent another paradigmatic class of inorganic nanomaterials in cancer nanomedicine, distinguished by their unique optical properties, ease of synthesis, and versatile surface chemistry. Owing to their localized surface plasmon resonance, gold nanoparticles efficiently convert light into heat, enabling photothermal therapy upon near-infrared irradiation [60]. This property has been extensively exploited for tumor ablation, often in combination with drug delivery or immunomodulatory approaches [69,70]. In addition, gold nanoparticles serve as robust platforms for drug conjugation, radiosensitization, and diagnostic imaging, as their surfaces can be readily functionalized with thiolated molecules, polymers, or biomolecules. Nevertheless, concerns regarding long-term biodistribution and persistence have motivated efforts to develop ultrasmall or biodegradable gold-based constructs to enhance renal clearance and translational feasibility [71,72].

Quantum dots (QDs) represent another important class of inorganic nanomaterials that have been widely explored for cancer imaging and theranostic applications. Their size-dependent optical properties, high photostability, and narrow emission spectra enable multiplexed imaging and real-time tracking of nanoparticle biodistribution in vivo. Early seminal studies demonstrated the strong potential of QD-based systems for tumor imaging and targeted delivery, although concerns regarding heavy-metal composition and long-term toxicity have motivated the development of biodegradable and cadmium-free alternatives [73,74,75].

**Figure 4:**
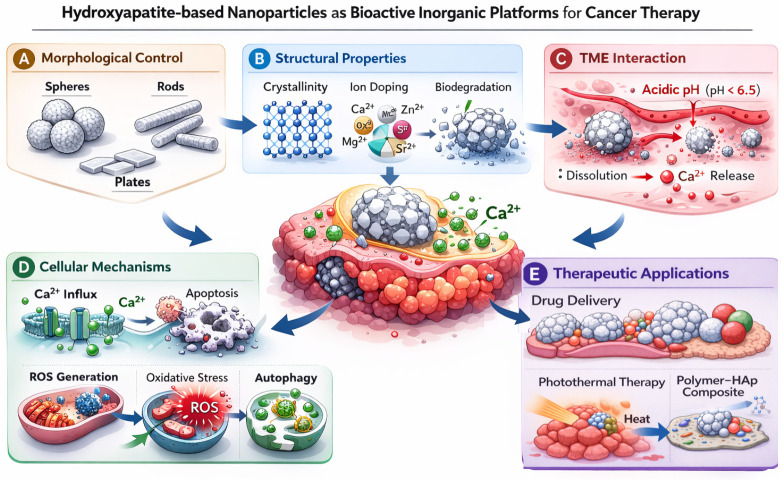
Hydroxyapatite-based nanoparticles as bioactive inorganic platforms for cancer therapy. Schematic representation of the multiscale design principles and therapeutic mechanisms of hydroxyapatite (HAp)-based nanoparticles in cancer applications: (**A**) Morphological control of HAp nanoparticles, including spherical, rod-like, and plate-like nanostructures, achieved through synthesis parameter tuning. (**B**) Structural and compositional features governing bioactivity, such as crystallinity, defect density, ionic substitution, and controlled biodegradation. (**C**) Interaction with the tumor microenvironment, where acidic pH conditions promote partial HAp dissolution and localized Ca^2+^ release. (**D**) Ion-mediated cellular mechanisms, including perturbation of intracellular calcium homeostasis, induction of oxidative stress, and activation of apoptotic or autophagic pathways in cancer cells. (**E**) Therapeutic applications of HAp-based nanoplatforms, encompassing drug delivery, hybrid polymer–HAp nanocomposites, and combination strategies integrating additional therapeutic modalities. This figure highlights the dual role of hydroxyapatite nanoparticles as both delivery vehicles and intrinsically bioactive materials, emphasizing how structure–property relationships underpin their biological and therapeutic performance.

Within this context, calcium phosphate-based inorganic nanoparticles, particularly hydroxyapatite (Ca_10_(PO_4_)_6_(OH)_2_)-based systems, have gained renewed attention as promising bioactive and biodegradable platforms for cancer therapy [61,76]. Hydroxyapatite is the principal mineral component of bone and teeth and has a long history of clinical use in orthopedic and dental applications, supporting its intrinsic biocompatibility and favorable safety profile [77]. Beyond regenerative medicine, hydroxyapatite nanoparticles and hydroxyapatite-based composites have been systematically investigated as tunable nanoscale systems with controllable morphology, surface chemistry, and degradation behavior, which collectively govern their biological performance [78,79]. Recent analyses have further highlighted hydroxyapatite-based nanomaterials as biobased platforms for cancer diagnosis and therapy, emphasizing their compositional versatility, intrinsic bioactivity, and compatibility with hybrid organic–inorganic designs [76]. The multiscale design principles governing hydroxyapatite-based nanoplatforms, from nanoscale composition to cellular and microenvironmental interactions, are schematically summarized in Figure 4.

Within this context, hybrid and composite nanoparticle systems have emerged as a versatile strategy to overcome the intrinsic limitations of single-material platforms. By combining complementary material classes within a single nanoscale construct, these systems aim to integrate distinct physicochemical, biological, and functional advantages while maintaining control over synthesis, stability, and performance.

Rather than categorizing hybrid systems using potentially ambiguous terminology, they can be conceptually grouped according to their dominant structural and functional design principles. First, soft-matter hybrid systems integrate multiple organic components—such as lipid–polymer or polymer–polymer combinations—to optimize colloidal stability, responsiveness to environmental cues, and drug encapsulation efficiency. Second, inorganic–organic composite systems couple the structural or physicochemical functionality of inorganic cores (e.g., magnetic, plasmonic, or bioactive materials) with the tunability and biocompatibility of organic coatings or matrices. Third, bioinspired hybrid platforms incorporate biological components—such as cell membranes, peptides, or extracellular vesicle elements—to exploit endogenous transport pathways and intercellular communication mechanisms.

Together, these strategies illustrate how hybridization enables increasingly sophisticated nanoparticle architectures capable of addressing the complex biological barriers and microenvironmental heterogeneity characteristic of solid tumors.

#### 3.3.1. Nano–Bio Interactions and Mechanistic Pathways

At the nano–bio interface, hydroxyapatite nanoparticles exhibit biological activities that extend well beyond their role as passive carriers. Cellular interactions are strongly influenced by particle size, crystallinity, aspect ratio, and surface chemistry, which determine protein adsorption, membrane affinity, and internalization pathways. Experimental studies have demonstrated that functionalized calcium phosphate and hydroxyapatite nanoparticles can be efficiently internalized by living cells, with intracellular localization governed by nanoscale structure and surface functionalization [80,81]. Internalization typically proceeds through endocytic mechanisms, followed by trafficking to acidic endo/lysosomal compartments, where partial dissolution of hydroxyapatite occurs.

Perturbation of intracellular calcium homeostasis represents a central mechanistic pathway underlying the biological effects of hydroxyapatite nanoparticles. Acid-triggered dissolution results in localized release of calcium and phosphate ions, which can disrupt mitochondrial membrane potential, enhance reactive oxygen species generation, and activate calcium-dependent signaling cascades associated with apoptosis or autophagy in cancer cells [82,83]. These ion-mediated effects are highly sensitive to material parameters, including particle size, crystallinity, and defect density, underscoring the importance of structure–property relationships in dictating biological outcomes.

#### 3.3.2. Advanced Design Strategies and Hybrid Systems

Building on these intrinsic mechanisms, advanced design strategies have expanded the functional landscape of hydroxyapatite-based nanoplatforms. Ionic substitution and doping with biologically active elements such as zinc, strontium, magnesium, selenium, or iron enable modulation of crystallinity, degradation kinetics, and biological response, while introducing additional therapeutic or diagnostic functionalities [84,85]. In parallel, hydroxyapatite nanoparticles have been explored as immunomodulatory agents capable of influencing inflammatory signaling, macrophage polarization, and tumor–immune interactions, further broadening their relevance for cancer nanomedicine [86,87].

Hybrid organic–inorganic systems represent a particularly powerful approach to exploit the multifunctionality of hydroxyapatite. Incorporation of hydroxyapatite nanoparticles into biodegradable polymeric matrices enables integration of intrinsic bioactivity with controlled degradation, mechanical stability, and drug delivery capability. Such hybrid designs have been demonstrated using hydroxyapatite-based nanoparticles embedded in polymeric scaffolds for controlled release applications, providing a transferable framework for therapeutic delivery concepts in oncology [88].

**Figure 5:**
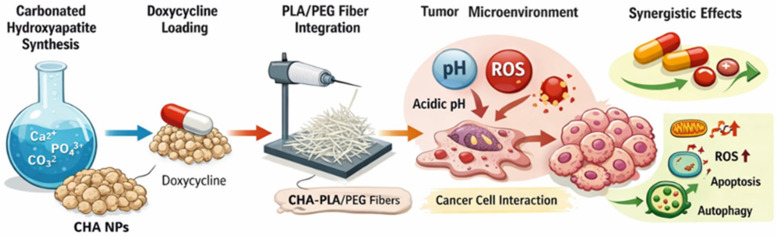
Schematic illustration of carbonated hydroxyapatite (CHA) nanoparticles loaded with doxycycline and incorporated into PLA/PEG fibrous scaffolds for cancer therapy. CHA nanoparticles exhibit pH-responsive dissolution and ion release under tumor-associated acidic conditions, while the fibrous polymeric matrix provides structural support and controlled drug delivery. The combined system promotes synergistic anticancer effects through enhanced intracellular drug release, modulation of calcium homeostasis, increased reactive oxygen species generation, and activation of apoptotic and autophagic pathways. Adapted from [89].

Representative examples include carbonated hydroxyapatite nanoparticles loaded with doxycycline and integrated into PLA/PEG fibrous scaffolds, where the inorganic phase contributes pH-responsive dissolution and ion-mediated biological effects, while the polymeric fibers provide structural integrity and sustained drug release. These hierarchically organized systems have been shown to exert synergistic anticancer effects in vitro, highlighting the potential of combining material-driven bioactivity with conventional chemotherapy [89]. The design rationale and proposed mechanism of action of such hybrid carbonated hydroxyapatite-based systems are schematically illustrated in Figure 5.

#### 3.3.3. Translational Perspective and Critical Positioning

From a translational perspective, hydroxyapatite-based nanoparticles occupy a unique position among inorganic nanomaterials. Unlike gold or iron oxide nanoparticles, which rely primarily on externally applied physical stimuli and may persist long term in biological tissues, hydroxyapatite degrades into physiologically regulated ions that are naturally processed by the body. This intrinsic biodegradability, together with extensive regulatory experience in calcium phosphate biomaterials, supports the translational attractiveness of hydroxyapatite-based platforms [77,82].

Taken together, the intrinsic biocompatibility, chemical similarity to native mineralized tissues, tunable nanoscale properties, and established clinical use of hydroxyapatite-based materials position these nanoparticles as one of the most realistic inorganic platforms for translational cancer nanomedicine, bridging mechanistic functionality with regulatory and manufacturing feasibility. Nevertheless, challenges related to reproducible nanoscale synthesis, batch-to-batch variability, and standardized physicochemical and biological characterization remain significant. Addressing these issues will be essential to advance hydroxyapatite nanoplatforms from mechanistic and preclinical studies toward clinically viable, mechanism-oriented cancer nanomedicines.

### 3.4. Bioinspired Nanoparticles

**Figure 6:**
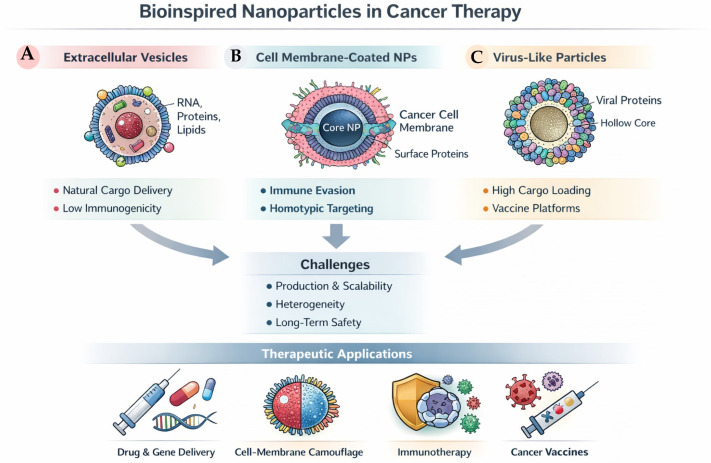
Bioinspired nanoparticle platforms for cancer therapy. Schematic overview of the main classes of bioinspired nanoparticle systems developed for cancer therapy and their representative biological functions: (**A**) Extracellular vesicles, including exosomes, highlighting their endogenous origin, natural cargo transport capabilities, and intrinsic roles in intercellular communication. (**B**) Cell membrane-coated nanoparticles, in which synthetic nanoparticle cores are cloaked with membranes derived from erythrocytes, immune cells, or cancer cells to confer immune evasion, prolonged circulation, and homotypic targeting properties. (**C**) Virus-like particles, retaining highly ordered viral capsid architectures while lacking infectious genetic material, enabling efficient cellular entry and antigen presentation. This figure illustrates how bioinspired design strategies exploit biological complexity to enhance nanoparticle–cell interactions, targeting specificity and therapeutic functionality while also highlighting the translational challenges associated with material heterogeneity and scalable manufacturing.

Bioinspired nanoparticles have emerged as a rapidly growing class of delivery systems that leverage principles derived from natural biological structures to improve biocompatibility, targeting, and functional integration with complex biological environments. Rather than constituting a single material category, bioinspired nanoplatforms encompass a diverse set of strategies that can be broadly grouped according to their biological source and mode of interaction with cells, including extracellular vesicles, cell membrane-coated nanoparticles, and virus-like particles. These platforms differ substantially in structural complexity, biological function, and translational maturity, providing complementary approaches to addressing delivery and targeting challenges in cancer therapy. The main classes of bioinspired nanoparticle platforms and their representative biological features and therapeutic functions are schematically summarized in Figure 6. Rather than relying solely on synthetic material optimization, these platforms seek to mimic or directly incorporate biological components such as cell membranes, extracellular vesicles, viruses, or endogenous macromolecules, thereby exploiting evolutionarily optimized pathways for cellular interaction and transport [86,87].

Among bioinspired systems, extracellular vesicles (EVs), including exosomes, have attracted particular attention due to their intrinsic role in intercellular communication. As naturally secreted nanoscale vesicles, extracellular vesicles exemplify bioinspired platforms that rely on endogenous biological pathways for cargo transport and cellular uptake, highlighting the potential advantages of leveraging native intercellular communication mechanisms. Exosomes are nanoscale vesicles secreted by most cell types and naturally carry proteins, lipids, and nucleic acids, enabling efficient cellular uptake and biological signaling. Their endogenous origin confers low immunogenicity and inherent targeting capabilities, which have been explored for the delivery of chemotherapeutic agents, RNA therapeutics, and immunomodulatory payloads in cancer therapy [90,91]. However, challenges related to scalable production, cargo loading efficiency, and heterogeneity remain significant obstacles to their clinical translation [92].

Cell membrane-coated nanoparticles represent another prominent bioinspired strategy, combining synthetic nanoparticle cores with membranes derived from erythrocytes, platelets, immune cells, or cancer cells. By transferring complex membrane-associated functionalities from source cells to synthetic cores, these systems illustrate how bioinspired design can endow nanoparticles with immune evasion, prolonged circulation, and selective cellular recognition without extensive chemical modification. This approach enables nanoparticles to inherit complex surface functionalities, including immune evasion, prolonged circulation, and homotypic targeting, without the need for extensive chemical functionalization [93,94]. Cancer cell membrane-coated nanoparticles, in particular, have been shown to exploit self-recognition mechanisms to enhance tumor accumulation, while immune cell-derived membranes can be used to modulate immune responses within the tumor microenvironment [95,96].

Virus-like particles (VLPs) constitute a further class of bioinspired nanoplatforms, retaining the highly ordered and repetitive structures of viral capsids while lacking infectious genetic material. These platforms exemplify structure-driven bioinspiration, in which precise nanoscale organization and repetitive architectures facilitate efficient cellular entry and immune engagement while minimizing the risks associated with viral replication. VLPs offer precise nanoscale architectures, high cargo loading capacity, and efficient cellular entry, making them attractive candidates for cancer vaccines and targeted delivery applications [97,98]. Nonetheless, pre-existing immunity, manufacturing complexity, and regulatory considerations must be carefully addressed for clinical translation.

Despite their promise, bioinspired nanoparticles also raise important challenges related to reproducibility, standardization, and safety. The diversity of bioinspired platforms, spanning endogenous vesicles, membrane-functionalized constructs, and virus-mimetic architectures, underscores the balance between biological sophistication and translational robustness that characterizes this class of nanomedicines. The complexity inherent to biological materials can result in batch-to-batch variability, incomplete characterization, and unpredictable in vivo behavior. Furthermore, the long-term fate and immunological consequences of repeated administration remain incompletely understood [3,12]. As a result, bioinspired nanomedicines exemplify the trade-off between biological sophistication and translational robustness, highlighting the need for integrative design frameworks that balance functionality with manufacturability and regulatory feasibility.

## 4. Targeting Strategies in Nanoparticle-Based Cancer Therapy

**Figure 7:**
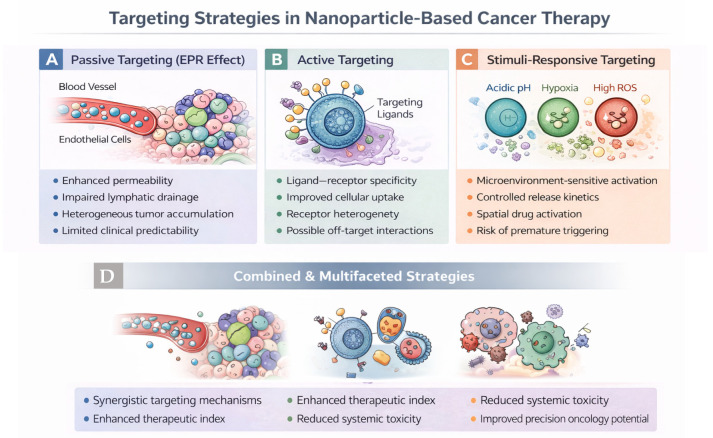
Targeting strategies in nanoparticle-based cancer therapy. Schematic representation of the main targeting strategies employed in nanoparticle-based cancer therapy: (**A**) Passive targeting, based on preferential nanoparticle accumulation in tumor tissue driven by abnormal vasculature and impaired lymphatic drainage. (**B**) Active targeting through ligand–receptor interactions, in which surface-functionalized nanoparticles engage overexpressed receptors on cancer or tumor-associated endothelial cells to promote receptor-mediated uptake. (**C**) Stimuli-responsive targeting, where physicochemical cues characteristic of the tumor microenvironment, such as acidic pH, enzymatic activity, hypoxia, or redox gradients, trigger nanoparticle activation or drug release. (**D**) Cellular and immune-mediated targeting strategies that exploit endogenous cell trafficking pathways, including immune cell homing and bioinspired transport mechanisms. This figure highlights the evolution of targeting concepts from passive accumulation models toward integrated, multilevel strategies that combine material design, tumor biology, and microenvironmental responsiveness.

Efficient targeting of nanoparticles to tumor tissues and cancer cells remains a central challenge in cancer nanomedicine. Beyond material composition, targeting strategies determine the spatial and cellular specificity of nanoparticle accumulation and therapeutic action. Targeting strategies in cancer nanomedicine can be broadly classified into passive, active, stimuli-responsive, and cellular or immune-mediated approaches, each addressing distinct biological barriers to effective tumor delivery. These strategies differ in their underlying mechanisms, degree of biological complexity, and translational maturity, ranging from vascular-level accumulation to receptor-specific uptake and microenvironment-triggered activation. An overview of the principal targeting paradigms and their representative mechanisms is schematically illustrated in Figure 7. These approaches aim to enhance tumor localization, minimize off-target effects, and improve therapeutic indices by exploiting physiological, molecular, or cellular differences between malignant and healthy tissues [3,99].

Beyond conventional ligand-based targeting, neuropeptide-functionalized nanoparticles have also demonstrated selective recognition of opioid receptors overexpressed in specific cancer cell types. In particular, neuropeptide–squalene nanoassemblies have shown efficient tumor targeting and improved therapeutic performance, highlighting how endogenous signaling pathways can be exploited to enhance specificity while preserving biocompatibility [100].

### 4.1. Passive Targeting and Its Limitations

Passive targeting, traditionally associated with the enhanced permeability and retention (EPR) effect, has long served as a foundational concept in cancer nanomedicine. Rather than representing a robust and universally applicable delivery mechanism, EPR-mediated nanoparticle accumulation is now recognized as a highly variable and context-dependent phenomenon, strongly influenced by tumor type, vascular architecture, stromal composition, and patient-specific factors [12,17]. As a result, passive targeting alone rarely ensures sufficient and homogeneous drug delivery to solid tumors in clinical settings.

From a transport perspective, nanoparticle extravasation and intratumoral distribution are governed not only by vascular permeability but also by interstitial fluid pressure, extracellular matrix density, and vascular functionality. These factors impose stringent constraints on nanoparticle size, deformability, and surface properties, often limiting deep tumor penetration even when vascular accumulation occurs. Consequently, EPR-driven delivery frequently results in perivascular localization rather than uniform distribution throughout the tumor mass.

Importantly, recent intravital imaging and quantitative studies have challenged simplified EPR-centric models by demonstrating that nanoparticle entry into tumors is frequently dominated by active transcytosis across endothelial cells rather than passive diffusion through inter-endothelial gaps [11]. This paradigm shift underscores that vascular biology and endothelial transport mechanisms play a central role in nanoparticle delivery and that passive accumulation cannot be assumed solely on the basis of nanoscale size or prolonged circulation.

Clinical meta-analyses have further reinforced these limitations, revealing that, on average, less than 1% of the administered nanoparticle dose reaches solid tumors, with substantial interpatient variability and poor predictability across tumor types [11,12]. These observations highlight a critical disconnect between preclinical EPR-based expectations and clinical performance, particularly in human tumors characterized by heterogeneous vasculature and dense stromal barriers.

Taken together, these findings indicate that passive targeting should be regarded as an enabling but insufficient mechanism for effective cancer nanomedicine. While prolonged circulation and favorable pharmacokinetics remain necessary prerequisites, they must be complemented by additional design strategies that actively promote tumor access, endothelial interaction, and cellular uptake. This recognition has driven the development of active targeting, stimuli-responsive systems, and multifaceted delivery strategies aimed at overcoming the intrinsic limitations of passive accumulation, as discussed in the following sections.

### 4.2. Active Targeting via Ligand–Receptor Interactions

Active targeting strategies aim to enhance the specificity of nanoparticle–cell interactions by functionalizing the nanoparticle surface with ligands that recognize receptors overexpressed on cancer cells or tumor-associated endothelial cells. Rather than substantially increasing total tumor accumulation, this approach primarily exemplifies receptor-mediated targeting, whereby molecular recognition is exploited to promote selective cellular uptake and intracellular delivery. Common targeting ligands include antibodies and antibody fragments, peptides, aptamers, sugars, and small molecules such as folate, which can trigger receptor-mediated endocytosis following ligand–receptor engagement [101,102].

A representative and instructive example is provided by transferrin-functionalized nanoparticles targeting the transferrin receptor (TfR), which is frequently overexpressed in rapidly proliferating cancer cells. While transferrin ligands effectively enhance receptor-mediated internalization at the cellular level, systematic studies have demonstrated that adsorption of plasma proteins and formation of a biomolecular corona can mask targeting ligands in vivo, thereby limiting improvements in overall tumor accumulation [15]. These findings highlight that ligand-mediated targeting primarily modulates intracellular trafficking and endocytic uptake rather than macroscopic tumor localization.

More recent strategies have sought to address these limitations through conditional or activatable targeting designs. For instance, nanoparticles equipped with pH-responsive or enzyme-cleavable shielding layers can remain “stealth” during circulation but expose targeting ligands selectively within the tumor microenvironment, where acidic pH or protease activity is prevalent. Such designs have been shown to enhance receptor accessibility and intracellular uptake while minimizing off-target interactions during systemic circulation [5,52]. These advances underscore the importance of coupling ligand–receptor recognition with microenvironment-responsive mechanisms.

Despite continued innovation, the clinical impact of active targeting remains constrained by biological barriers, including heterogeneous receptor expression, limited tissue penetration, and dynamic protein corona formation [15,103]. Consequently, active targeting is increasingly viewed not as a means to dramatically enhance bulk tumor accumulation but rather as a strategy to improve cellular specificity, intracellular delivery, and therapeutic index when integrated with complementary delivery mechanisms.

### 4.3. Stimuli-Responsive and Microenvironment-Triggered Targeting

Stimuli-responsive targeting strategies exploit distinctive physicochemical and biological features of the tumor microenvironment to achieve spatially and temporally controlled activation of nanoparticle function. Rather than relying solely on constitutive ligand–receptor interactions, these systems are typically engineered to remain relatively inert during systemic circulation and undergo structural, chemical, or functional transformations in response to tumor-associated stimuli, thereby enhancing therapeutic precision while minimizing off-target effects [55,56].

Among endogenous tumor-associated triggers, acidic pH, redox gradients, and enzyme activity have been most extensively explored. Mild extracellular acidosis and pronounced endo/lysosomal acidification can induce protonation-driven destabilization of ionizable polymers or cleavage of acid-labile linkers, enabling selective drug release and/or exposure of functional motifs at the tumor site [55,56]. Similarly, elevated intracellular glutathione levels can promote reductive cleavage of disulfide bonds, facilitating preferential intracellular cargo release following uptake, while enzyme-responsive designs can leverage tumor-associated proteases to activate delivery or unmask ligands locally [52,104].

Importantly, stimuli-responsive designs offer a practical route to mitigate limitations observed in passive and active targeting. By decoupling circulation stability from tumor-specific activation, responsive nanoparticles can reduce premature leakage, limit nonspecific interactions shaped by the protein corona, and improve intracellular delivery efficiency when combined with receptor-mediated uptake [55]. In this sense, responsiveness is increasingly viewed not as an isolated feature but as an enabling layer that can enhance the performance of other targeting modalities [35].

Recent advances have further emphasized multistage and hierarchical activation concepts, in which nanoparticles sequentially respond to multiple stimuli encountered during vascular transport, tissue penetration, cellular uptake, and intracellular trafficking. For example, systems combining pH-responsive shielding with enzyme-triggered ligand exposure or redox-sensitive intracellular release have been developed to increase receptor accessibility while preserving systemic stealth, thereby improving selectivity and therapeutic index in preclinical models [52,104]. These multilevel designs reflect a shift toward adaptive nanomedicines that respond dynamically to biological context rather than relying on a single static targeting mechanism.

Overall, stimuli-responsive targeting represents a key evolution in nanoparticle design, transforming nanocarriers from passive vehicles into dynamic systems that actively engage pathological cues within tumors. When integrated with optimized pharmacokinetics and complementary targeting strategies, microenvironment-activated nanoparticles provide a rational framework to address the heterogeneity and biological complexity that limit conventional cancer nanomedicine [55,56].

### 4.4. Cellular and Immune-Mediated Targeting

Beyond molecular recognition and physicochemical responsiveness, emerging targeting strategies increasingly seek to exploit endogenous cellular and immune mechanisms to guide nanoparticle delivery. These approaches rely on intrinsic trafficking, homing, and communication pathways inherent to immune and stromal cells, representing a higher level of biointegration than purely material-driven targeting strategies. Rather than directing nanoparticles toward tumors through engineered surface motifs alone, cellular and immune-mediated targeting leverages biological processes that have evolved to navigate complex tissue environments.

Immune cell-mediated delivery constitutes a prominent example of this paradigm. Macrophages, neutrophils, and T cells exhibit natural tumor-homing capabilities driven by inflammatory cues and chemokine gradients and have been explored as cellular carriers for nanoparticle payloads. Nanoparticles internalized or associated with these cells can be transported across vascular and stromal barriers, enabling access to poorly perfused tumor regions that are otherwise difficult to reach through conventional delivery mechanisms. Such strategies highlight the potential of immune cells to function as active vectors rather than passive bystanders in nanoparticle transport.

In parallel, bioinspired platforms such as cell membrane-coated nanoparticles and extracellular vesicles incorporate complex and multifunctional targeting motifs derived directly from biological sources [90,93]. By preserving membrane proteins, lipids, and glycans from their parent cells, these systems inherently encode immune evasion, homotypic targeting, and intercellular communication capabilities that are difficult to replicate synthetically. As a result, cellular mimicry enables selective interactions with tumor cells, immune components, and the tumor microenvironment without relying on single-ligand targeting paradigms.

Importantly, cellular and immune-mediated targeting strategies shift the design focus from static nanoparticle properties toward dynamic, systems-level interactions within the tumor ecosystem. While these approaches offer unique opportunities to overcome vascular, stromal, and cellular barriers, they also introduce new challenges related to safety, control over biodistribution, scalability, and reproducibility. Ensuring consistent biological composition, minimizing unintended immune activation, and achieving robust manufacturing standards remain critical hurdles for clinical translation.

Overall, cellular and immune-mediated targeting represents a conceptual expansion of nanoparticle design, emphasizing cooperation with biological systems rather than purely engineered solutions. When integrated with controlled material design and responsive activation mechanisms, these biointegrated strategies provide a promising pathway toward more effective and adaptive cancer nanomedicine.

### 4.5. Outlook on Targeting Strategies

Collectively, targeting strategies in cancer nanomedicine have evolved from simplistic passive accumulation models toward multifaceted approaches that integrate material design, tumor biology, and microenvironmental cues. Together, these complementary paradigms illustrate the multilevel nature of nanoparticle–tumor interactions, spanning vascular transport, molecular recognition, microenvironmental responsiveness, and cellular trafficking, as summarized in Figure 7. Rather than relying on a single targeting mechanism, increasing evidence suggests that effective tumor targeting will require combinatorial strategies that address vascular transport, tissue penetration, cellular uptake, and intracellular trafficking simultaneously [3,4].

In this context, targeting should be viewed not as an isolated design feature but as an emergent property of the dynamic nano–bio interface, where the interplay of engineered nanomaterials and complex biological systems leads to enhanced therapeutic outcomes. Future advancements will likely focus on the integration of real-time monitoring and adaptive delivery, leveraging advances in imaging, biomarker identification, and machine learning to tailor nanoparticle behavior in vivo. By embracing a holistic and adaptive approach, the field can move beyond static designs toward truly personalized and effective nanomedicine.

An illustrative example of these advancements is the development of stimuli-responsive nanoparticles capable of adapting to multiple tumor-associated cues, such as acidic pH, enzymatic activity, or redox gradients, enabling spatially and temporally controlled drug activation within the tumor microenvironment [3,55]. These nanoparticles are engineered to remain stable in circulation and to activate drug release selectively within the tumor, responding dynamically to the local conditions. This level of sophistication allows for precise, on-demand therapy, minimizing off-target effects and improving therapeutic efficacy.

## 5. Tumor Microenvironment Modulation by Nanoparticles

The tumor microenvironment (TME) plays a central and active role in cancer progression, therapeutic resistance, and immune evasion, extending far beyond a passive backdrop for malignant cell growth. In addition to cancer cells, tumors comprise a heterogeneous and dynamically evolving ecosystem that includes stromal fibroblasts, diverse immune cell populations, extracellular matrix (ECM) components, and aberrant vascular networks, together with distinct physicochemical conditions such as hypoxia, acidity, and elevated interstitial fluid pressure. These features not only limit the efficacy of conventional therapies but also actively shape treatment response and disease evolution, thereby representing actionable intervention points for nanoparticle-based strategies [105,106].

**Figure 8:**
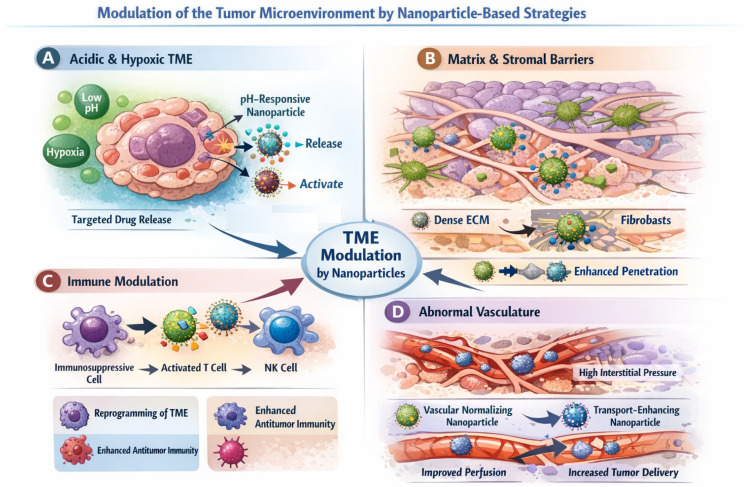
Modulation of the tumor microenvironment by nanoparticle-based strategies. Schematic illustration of the major biological components of the tumor microenvironment (TME) and representative nanoparticle-based approaches designed to modulate tumor-associated barriers and enhance therapeutic efficacy. (**A**) Physicochemical features of the TME, including acidic pH and hypoxic conditions, which can be exploited by pH-responsive and hypoxia-activated nanoparticles to achieve selective and localized drug release. (**B**) Extracellular matrix and stromal barriers, where matrix-degrading or stroma-targeting nanoparticles facilitate enhanced tissue penetration and improved intratumoral access. (**C**) Immune modulation within the TME, highlighting nanoparticle-mediated reprogramming of immunosuppressive cell populations and activation of antitumor immune responses. (**D**) Abnormal tumor vasculature and interstitial transport, illustrating nanoparticle strategies aimed at vascular normalization, improved perfusion, and enhanced transport into tumor tissue. Together, these approaches illustrate how rational nanoparticle design can be leveraged to actively remodel the tumor microenvironment, thereby overcoming biological resistance mechanisms and improving therapeutic outcomes.

In contrast to targeting strategies that primarily aim to enhance nanoparticle access to tumor tissue (as discussed in Section 4), TME modulation focuses on actively reshaping the biological and physical context in which therapies operate. Nanoparticles offer unique opportunities to intervene at multiple levels of the tumor ecosystem, enabling localized and temporally controlled modulation of physicochemical conditions, stromal architecture, immune responses, and vascular function. The major tumor-associated barriers and the corresponding nanoparticle-enabled strategies designed to remodel these components are schematically summarized in Figure 8.

### 5.1. Physicochemical Features of the Tumor Microenvironment

Solid tumors exhibit pronounced physicochemical heterogeneity, characterized by acidic extracellular pH, spatially heterogeneous hypoxia, and altered redox gradients. These conditions arise from elevated glycolytic metabolism, impaired lymphatic drainage, and disorganized vasculature that limits efficient oxygen and nutrient delivery. As tumors grow and evolve, steep gradients in pH, oxygen tension, and metabolic byproducts develop, contributing to regional therapeutic resistance and phenotypic heterogeneity within the tumor mass.

Rather than merely serving as passive constraints, these physicochemical features constitute powerful design inputs for nanoparticle engineering. By exploiting tumor-associated acidity or hypoxia, nanocarriers can be programmed to undergo conditional activation, structural reconfiguration, or site-specific payload release. Such designs enable therapeutic activation that is spatially confined to tumor tissue and decoupled from systemic circulation, thereby improving selectivity and reducing off-target toxicity in healthy tissues [55,107]. Beyond drug release, pH- and hypoxia-responsive nanoparticles can also modulate intracellular trafficking, endosomal escape, and metabolic stress responses, further amplifying therapeutic impact.

Importantly, physicochemical modulation is increasingly being combined with other design axes, such as immune activation or stromal remodeling, highlighting the potential of nanoparticles to orchestrate coordinated microenvironmental interventions rather than isolated responses.

### 5.2. Extracellular Matrix and Stromal Barriers

The tumor extracellular matrix (ECM) constitutes a dominant physical and biochemical barrier to effective nanoparticle transport and homogeneous intratumoral distribution. Excessive deposition of fibrillar collagen, elevated hyaluronic acid content, and increased matrix crosslinking collectively result in enhanced tissue stiffness, reduced interstitial porosity, and elevated solid stress. These features severely constrain nanoparticle diffusion and convective transport, promoting perivascular drug accumulation and leaving poorly perfused tumor regions therapeutically underserved.

Beyond its structural role, the tumor stroma—particularly cancer-associated fibroblasts (CAFs)—actively regulates ECM composition, mechanical properties, and remodeling dynamics. Through secretion of matrix components, crosslinking enzymes, and paracrine signaling factors, CAFs reinforce transport barriers and foster resistance to both cytotoxic and immune-based therapies. Consequently, nanoparticle strategies designed to interact with, remodel, or transiently disrupt stromal barriers have gained increasing attention.

Approaches including enzymatic matrix degradation, targeting of ECM components, or functional reprogramming of stromal cells have demonstrated the capacity to enhance nanoparticle penetration, improve spatial drug distribution, and normalize mechanical stresses within tumor tissue [21,23]. Importantly, controlled and localized ECM modulation via nanoparticles enables transient barrier relaxation without the systemic toxicities associated with global stromal depletion, underscoring the advantages of nanomedicine-based intervention.

### 5.3. Immune Modulation Within the Tumor Microenvironment

The tumor microenvironment is frequently dominated by immunosuppressive signaling networks that impair effective antitumor immunity. This hostile immune landscape is characterized by the accumulation of regulatory T cells (Tregs), myeloid-derived suppressor cells (MDSCs), and tumor-associated macrophages (TAMs) skewed toward pro-tumorigenic phenotypes, alongside impaired dendritic cell maturation and dysfunctional cytotoxic T lymphocyte activity. Collectively, these features contribute to immune evasion, tumor progression, and resistance to immunotherapy.

Nanoparticle-based delivery systems provide a versatile platform to locally reshape immune signaling within the TME with enhanced spatial and temporal precision. Through selective delivery of immunostimulatory agents, cytokines, nucleic acids, or metabolic modulators, nanoparticles can reprogram suppressive immune populations, enhance antigen presentation, and restore effector immune cell activity directly within tumor tissue. Importantly, nanoparticle-mediated co-delivery of tumor antigens and immune adjuvants enables coordinated immune priming while limiting systemic exposure and immune-related adverse effects.

These immunomodulatory strategies have gained increasing relevance in combination with immune checkpoint inhibitors, where nanoparticle-enabled remodeling of the TME has shown promise in sensitizing immunologically “cold” tumors and overcoming therapeutic resistance. By enabling localized immune activation and reducing systemic toxicity, nanomedicine-based approaches provide a powerful framework for synergistic and more durable cancer immunotherapy outcomes [108,109,110,111,112,113].

### 5.4. Vascular Normalization and Interstitial Transport

The aberrant architecture and functionality of tumor vasculature represent major determinants of inefficient drug delivery within solid tumors. Disorganized vessel networks, irregular blood flow, and excessive vascular permeability collectively result in heterogeneous perfusion, elevated interstitial fluid pressure, and compromised convective and diffusive transport. These abnormalities not only limit nanoparticle penetration but also hinder immune cell infiltration and spatially uniform therapeutic exposure.

Nanoparticle-based strategies have therefore evolved beyond passive exploitation of vascular leakiness toward active modulation of vascular and interstitial transport properties. Nanocarriers capable of delivering vascular-normalizing agents, regulating angiogenic signaling, or responding dynamically to hemodynamic and microenvironmental cues can transiently restore vascular function, reduce interstitial pressure, and enhance tissue perfusion. Such interventions improve the intratumoral distribution of both nanomedicines and immune effector cells, amplifying therapeutic efficacy and supporting combination treatment paradigms [17,22,114,115,116,117].

Together with physicochemical targeting, ECM remodeling, and immune modulation, vascular normalization represents a complementary and synergistic design axis for next-generation nanotherapeutics. Collectively, these four strategies illustrate how rational nanoparticle engineering can be strategically aligned with distinct tumor microenvironmental features to overcome biological transport barriers and achieve more effective and durable therapeutic outcomes.

## 6. Nanoparticles in Cancer Immunotherapy

Cancer immunotherapy has fundamentally reshaped the therapeutic landscape by leveraging the immune system’s capacity to recognize and eliminate malignant cells. Immune checkpoint inhibitors, adoptive cell therapies, cancer vaccines, and cytokine-based interventions have demonstrated durable clinical responses in selected patient populations. However, limited response rates, immune-related adverse events, and the emergence of adaptive resistance mechanisms continue to constrain their broader clinical impact. In this context, nanoparticle-based delivery systems have emerged not merely as passive carriers but as active modulators of immune activation, offering new opportunities to enhance efficacy, specificity, and safety across multiple immunotherapeutic modalities [108,109].

**Figure 9:**
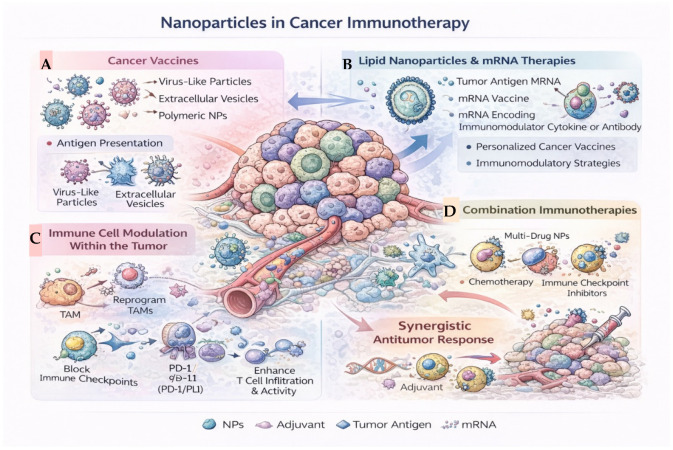
Nanoparticle-based strategies in cancer immunotherapy. Schematic overview of the principal roles of nanoparticles in cancer immunotherapy and their interactions with immune components: (**A**) Nanoparticle-based cancer vaccines enabling the delivery of tumor-associated antigens, neoantigens, and adjuvants to antigen-presenting cells, promoting efficient antigen processing and T cell priming. (**B**) Lipid nanoparticle (LNP) platforms for mRNA-based immunotherapies, facilitating transient expression of tumor antigens, cytokines, or immune-modulatory proteins with controlled biodistribution and immunogenicity. (**C**) Modulation of immune cells within the tumor microenvironment, including reprogramming of tumor-associated macrophages and enhancement of cytotoxic T cell activity through localized nanoparticle delivery. (**D**) Combination immunotherapeutic strategies, in which nanoparticles enable the spatial and temporal coordination of immunotherapy with chemotherapy, radiotherapy, or targeted therapies. This figure highlights how nanoparticle engineering can be leveraged to enhance immune activation, improve therapeutic specificity, and reduce systemic toxicity in cancer immunotherapy.

Rather than functioning through a single mechanism, nanoparticles enable multilevel immune engagement by controlling antigen presentation, modulating immune cell phenotypes, coordinating spatial and temporal immune activation, and enabling rational combination therapies. The principal nanoparticle-enabled immunotherapeutic strategies—including vaccine delivery, mRNA-based platforms, immune cell modulation, and combination approaches—are schematically summarized in Figure 9.

### 6.1. Nanoparticle-Based Cancer Vaccines

Nanoparticles have been extensively investigated as platforms for cancer vaccine delivery owing to their ability to efficiently transport tumor-associated antigens, neoantigens, and immunological adjuvants to antigen-presenting cells (APCs). By tailoring size, surface charge, and composition, nanocarriers can promote lymphatic drainage, enhance uptake by dendritic cells, and facilitate antigen cross-presentation via major histocompatibility complex class I pathways. These features address key limitations of soluble vaccine formulations, including poor stability, inefficient APC targeting, and suboptimal immune priming.

Both synthetic nanoparticles and bioinspired systems—such as virus-like particles and extracellular vesicles—have demonstrated strong immunogenic potential in preclinical models by mimicking pathogen-associated structural cues and enhancing innate immune activation [118,119]. Importantly, nanoparticle-based vaccines enable modular design, allowing antigens and adjuvants to be co-delivered in defined ratios and spatial arrangements, thereby improving the quality and durability of antitumor immune responses.

### 6.2. Lipid Nanoparticles and mRNA-Based Immunotherapies

Lipid nanoparticles (LNPs) have emerged as the leading platform for nucleic acid delivery, as highlighted by the rapid clinical translation of mRNA vaccines. In the context of cancer immunotherapy, mRNA–LNP systems enable transient, controllable expression of tumor antigens, cytokines, or immune-modulatory proteins, offering precise temporal regulation of immune activation. Early clinical studies demonstrating systemic RNA delivery to dendritic cells provided foundational proofs-of-concept for mRNA-based cancer vaccines and immunotherapies [120].

The modular architecture of LNPs allows fine-tuning of biodistribution, cellular targeting, and innate immune stimulation, making them particularly attractive for personalized immunotherapy strategies based on patient-specific neoantigens. Moreover, advances in ionizable lipid chemistry and formulation design have enabled improved endosomal escape and reduced systemic toxicity, further enhancing clinical feasibility [37,38]. As a result, mRNA–LNP platforms are increasingly viewed as flexible immunotherapy backbones rather than single-purpose delivery vehicles [3,44].

### 6.3. Modulation of Immune Cells Within the Tumor Microenvironment

Beyond antigen delivery, nanoparticles can be engineered to directly modulate immune cell function within the tumor microenvironment. Localized nanoparticle delivery enables selective reprogramming of immunosuppressive cell populations, such as tumor-associated macrophages, shifting them toward pro-inflammatory and antitumor phenotypes. Similarly, nanoparticles can facilitate intratumoral delivery of immune checkpoint inhibitors, cytokines, or nucleic acids, enhancing immune activation while limiting systemic exposure and associated toxicities. These approaches align closely with emerging concepts in tumor microenvironment remodeling, where localized immune modulation is leveraged to overcome resistance and enhance therapeutic responsiveness [106,109]. Importantly, nanoparticle-mediated immune reprogramming enables spatiotemporal control that is difficult to achieve with systemic immunotherapies, positioning nanomedicine as a key enabler of precision immuno-oncology.

### 6.4. Combination Strategies and Future Perspectives

The full therapeutic potential of nanoparticle-enabled immunotherapy is increasingly realized through combination strategies that integrate immunotherapy with chemotherapy, radiotherapy, or targeted therapies. Nanoparticles provide a unique opportunity to co-deliver multiple agents with complementary mechanisms of action, synchronize their pharmacokinetics, and spatially coordinate immune activation with tumor cell killing. Such coordination is particularly relevant for overcoming immunological tolerance and converting immunologically “cold” tumors into responsive disease states.

Despite promising preclinical outcomes, challenges related to immune toxicity, manufacturing complexity, regulatory standardization, and patient stratification remain critical considerations for clinical translation [3,4]. Addressing these challenges will require not only advances in nanoparticle engineering but also improved biomarker-driven patient selection and integration with systems-level immunological profiling. Collectively, these efforts position nanoparticle-based immunotherapy as a central component of next-generation combination cancer treatments.

## 7. Safety, Toxicity, and Clinical Translation of Nanoparticle-Based Cancer Therapies

Despite decades of intensive research and a growing body of promising preclinical data, the clinical translation of nanoparticle-based cancer therapies remains comparatively limited. The safety profile of nanoparticle-based therapeutics is highly context-dependent and influenced by material composition, size distribution, surface chemistry, dose, administration route, and tumor model. Reported half maximal inhibitory concentration (IC50) values for cancer cells vary widely across nanoparticle platforms and experimental conditions, limiting direct cross-comparisons. For example, iron oxide nanoparticles employed for magnetic hyperthermia typically exhibit IC50 values in the high microgram per milliliter range in vitro, whereas drug-loaded lipid or polymeric nanoparticles often demonstrate IC50 values comparable to their free-drug counterparts, depending on encapsulation efficiency and release kinetics [121]. Moreover, only a small fraction of systemically administered nanoparticles reaches tumor tissue in vivo, which further complicates the interpretation of in vitro potency data [12]. Importantly, cytotoxicity toward non-malignant cells frequently differs from cancer cell IC50 values, emphasizing the need to evaluate therapeutic index, biodistribution, and immune interactions rather than relying exclusively on in vitro cytotoxicity metrics [122]. These considerations highlight that translational safety assessment must integrate dose–response relationships, long-term accumulation, and immunological effects in addition to conventional cytotoxicity assays. While numerous nanomedicines have entered clinical trials and a subset has achieved regulatory approval, discrepancies between preclinical efficacy and clinical outcomes continue to challenge the field. These translational gaps highlight the need to move beyond proof-of-concept demonstrations toward a deeper understanding of safety, toxicity, and biological mechanisms that govern in vivo performance [4,12].

A central determinant of nanomedicine safety is biodistribution and long-term fate. Nanoparticle accumulation in off-target organs—particularly the liver, spleen, and lungs—remains a major concern, as these tissues are central to clearance via the mononuclear phagocyte system. Physicochemical parameters such as particle size, shape, surface chemistry, and protein corona formation strongly influence circulation time, organ sequestration, and elimination routes. In addition, incomplete biodegradation or slow clearance of certain inorganic and polymeric nanomaterials may result in prolonged tissue retention, raising concerns regarding chronic exposure and delayed toxicity. These issues underscore the importance of systematic evaluation of nanoparticle persistence, degradation products, and long-term biological interactions [10,61].

Interactions between nanoparticles and the immune system represent another critical safety dimension. While immune engagement is a desired feature in immunotherapeutic applications, unintended immune activation can lead to adverse effects such as complement activation-related pseudoallergy (CARPA), cytokine release syndrome, or immunosuppression. Importantly, subtle variations in nanoparticle composition, surface functionalization, or formulation protocols can profoundly alter immunological outcomes, complicating reproducibility and safety assessment. These observations emphasize the need for standardized immunotoxicity testing frameworks and predictive in vitro–in vivo models capable of capturing clinically relevant immune responses [123,124].

Beyond biological considerations, manufacturing scalability and reproducibility remain major bottlenecks for clinical translation. Many advanced nanoplatforms rely on complex multicomponent architectures, biological materials, or finely tuned surface modifications that are challenging to reproduce consistently at an industrial scale. Batch-to-batch variability can directly impact biodistribution, efficacy, and safety, complicating regulatory evaluation. Moreover, the absence of fully harmonized standards for nanoparticle characterization, stability testing, and quality control further hinders regulatory approval. Addressing these challenges requires early integration of regulatory considerations, adoption of good manufacturing practice (GMP)-compatible processes, and simplification of nanoparticle designs without compromising therapeutic function [125,126].

Encouragingly, there is a growing shift toward translationally robust nanomedicine design principles that prioritize clinical feasibility alongside biological performance. These approaches emphasize the use of biodegradable and clinically validated materials, streamlined architectures, and mechanisms of action that can be clearly linked to measurable biological outcomes. Integrating insights from tumor biology, immunology, and pharmacology—together with predictive modeling and clinically relevant animal models—will be essential to bridge the gap between laboratory innovation and patient benefit. In this context, limited clinical success should not be interpreted as a failure of nanotechnology itself but rather as a call for more biologically informed, standardized, and clinically grounded nanomedicine development strategies [3,4].

## 8. Conclusions and Future Perspectives

**Figure 10:**
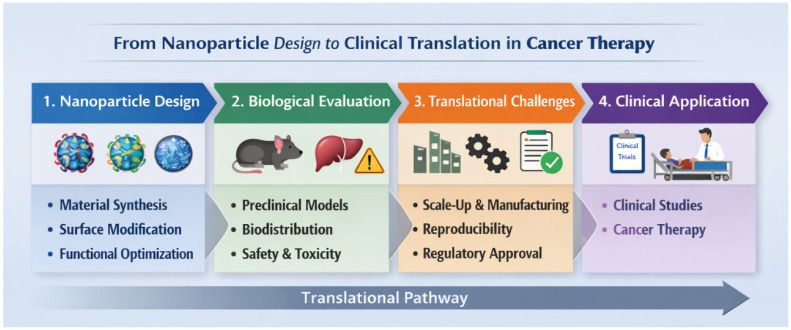
From nanoparticle design to clinical translation in cancer therapy. Conceptual overview summarizing the multiscale progression of nanoparticle-based cancer therapies from rational material design to clinical application. The figure integrates key elements discussed throughout this review, including nanoparticle platforms and physicochemical properties, targeting strategies, interactions with the tumor microenvironment, immune modulation, and translational considerations such as safety, manufacturability, and regulatory pathways. This schematic highlights the need for biology-guided and clinically informed nanoparticle design to bridge the gap between preclinical innovation and therapeutic impact in patients.

Nanoparticle-based strategies have reshaped the conceptual and technological landscape of cancer therapy, offering unprecedented opportunities to integrate material science, tumor biology, and immunology into multifunctional therapeutic platforms. As discussed throughout this review, the multilevel progression from nanoparticle design through biological interaction to clinical translation highlights the need for integrative and biologically grounded design frameworks rather than isolated material optimization. This progression is schematically summarized in Figure 10.

As discussed throughout this review, advances in nanoparticle design have enabled increasingly precise control over biodistribution, targeting, cargo delivery, and interaction with the tumor microenvironment. These developments have moved the field beyond simple carrier-based paradigms toward systems capable of actively engaging biological processes at multiple spatial and temporal scales.

Despite this progress, the clinical impact of cancer nanomedicine remains more limited than initially anticipated. The translational bottleneck is not primarily a consequence of insufficient technological sophistication but rather a consequence of an incomplete alignment between nanoparticle design and the biological complexity of human tumors. Heterogeneity in vascular architecture, extracellular matrix composition, immune landscapes, and interpatient variability continues to challenge generalized delivery strategies. In this context, the future of nanomedicine lies not in increasing structural complexity per se but in developing biologically informed, mechanism-driven designs that address specific barriers to therapeutic efficacy.

Emerging trends point toward several converging directions. First, simplification and standardization of nanoparticle architectures, favoring biodegradable materials and clinically validated components, are likely to enhance reproducibility and regulatory acceptance. Second, the integration of stimuli-responsive and microenvironment-adaptive features offers a rational means to achieve spatial and conditional specificity without reliance on overly complex targeting schemes. Third, bioinspired and immune-interfacing nanoplatforms are poised to play an increasingly important role, particularly in the context of cancer immunotherapy and combination treatment strategies.

Equally important is the need for improved preclinical models and translational frameworks. Conventional animal models often fail to recapitulate the physiological, immunological, and transport-related constraints present in human tumors, leading to overestimation of therapeutic benefit. Incorporating predictive modeling, human-relevant in vitro systems, and clinically meaningful endpoints will be essential to bridge the gap between laboratory innovation and patient outcomes. In parallel, early consideration of manufacturing scalability, quality control, and regulatory requirements should become integral components of nanoparticle design rather than downstream considerations.

In this regard, the growing maturity of inorganic and hybrid nanoparticle platforms—particularly bioactive systems such as hydroxyapatite-based nanomaterials—illustrates how rational material selection can directly contribute to biological function, safety, and translational relevance. Importantly, these systems exemplify materials whose clinical familiarity and degradation pathways align naturally with regulatory and safety expectations. When combined with responsive design principles, immune modulation, and microenvironment-aware strategies, these platforms exemplify a new generation of nanomedicines in which material properties, biological interaction, and therapeutic intent are intrinsically linked rather than sequentially optimized.

Overall, the evolution of cancer nanomedicine increasingly demonstrates that therapeutic success cannot be achieved through incremental optimization of nanoparticle physicochemical properties alone. Continued reliance on passive accumulation, oversimplified targeting paradigms, and one-size-fits-all carrier designs has proven insufficient to overcome the biological complexity and heterogeneity of solid tumors.

Moving forward, the field must shift away from designing ever more complex nanostructures without clear biological justification and instead prioritize mechanism-driven, biologically informed, and context-aware nanoparticle systems. Embracing tumor heterogeneity, integrating responsiveness to microenvironmental cues, and aligning material selection with translational feasibility should become central design principles rather than secondary considerations. Looking forward, several strategic action lines emerge for the field of cancer nanomedicine. First, future research should prioritize biologically validated design principles over incremental material modifications that lack mechanistic justification. Second, greater emphasis must be placed on reproducibility, scalable manufacturing, and critical quality attributes to facilitate clinical translation. Third, integration of advanced preclinical models, including organoids and immune-competent systems, is essential to better predict human responses. Finally, systematic correlation between nanoparticle physicochemical parameters and in vivo biological performance—supported by quantitative and data-driven approaches—will be crucial to move the field beyond empirical optimization toward predictive nanomedicine design. Ultimately, the future impact of cancer nanomedicine will depend on its ability to couple material innovation with a deep biological understanding and realistic clinical pathways.

## Data Availability

No new data were created or analyzed in this study. Data sharing is not applicable to this article.
